# Supplementary material for: Estimation of Regional Economic Development Indicator from Transportation Network Analytics
Source: Sci Rep. 2020 Feb 14;10:2647. doi: 10.1038/s41598-020-59505-2 (PMC7021790; doi:10.1038/s41598-020-59505-2)
Supplement: Supplementary file 1 — Supplementary information. [file 41598_2020_59505_MOESM1_ESM.pdf]

Appendix

Supplementary Document for "Estimation of Regional Economic Development Indicator from Transportation Network Analytics", *Scientific Reports.* https://doi.org/10.1038/s41598-020-59505-2

Bin Li[1], Song Gao[2]∗, Yunlei Liang[2], Yuhao Kang[2], Timothy Prestby[2], Yuqi Gao[2], Runmou Xiao[1]
1. School of Automobile, Chang'an University, Xi'an, Shaanxi, 710064, China
2. Geospatial Data Science Lab, Department of Geography, University of Wisconsin-Madison, WI, 53706, USA
*Corresponding author email: song.gao@wisc.edu

Modeling the GDP distribution

The empirical histogram distribution of the economic development indicator (GDP) values across all cities in the study period is a positively skewed distribution with a long-tail end (Fig. S1a). By fitting the data observations into four theoretical models (i.e., normal, gamma, log-normal, and Weibull distributions) using the maximum likelihood estimation[71], the results show that the log-normal model is the best with the maximum log-likelihood of -994.39 and the minimum Akaike information criterion (AIC) value of 1992.78. AIC is used to determine which model performs the best regarding the goodness of fit and the simplicity of a model using information theory[72]. Meanwhile, the log-likelihood and AIC values for other models are gamma distribution (log-likelihood=-1002.82 & AIC=2009.64), Weibull distribution (log-likelihood=-1004.23 & AIC=2012.45), and normal distribution (log-likelihood=-1066.17 & AIC=2136.35) respectively. Fig. S1b and Fig. S1d show the curves of empirical and theoretical cumulative distribution functions of these models and their quantile-quantile plots. The skewness-kurtosis plot (i.e., the Cullen and Frey graph[73]) is used to examine the characteristics of the skewness and the degree of tailedness (i.e., kurtosis) of the GDP data with uncertainty compared with the theoretical models. As shown in the S1c, the bootstrapping values fit better with the log-normal, gamma and Weibull models rather than the normal, uniform and logistic models. The consistent positive skewness and kurtosis values verify the "heavy-tailed on the right" characteristic of the city GDP distribution in the study areas.

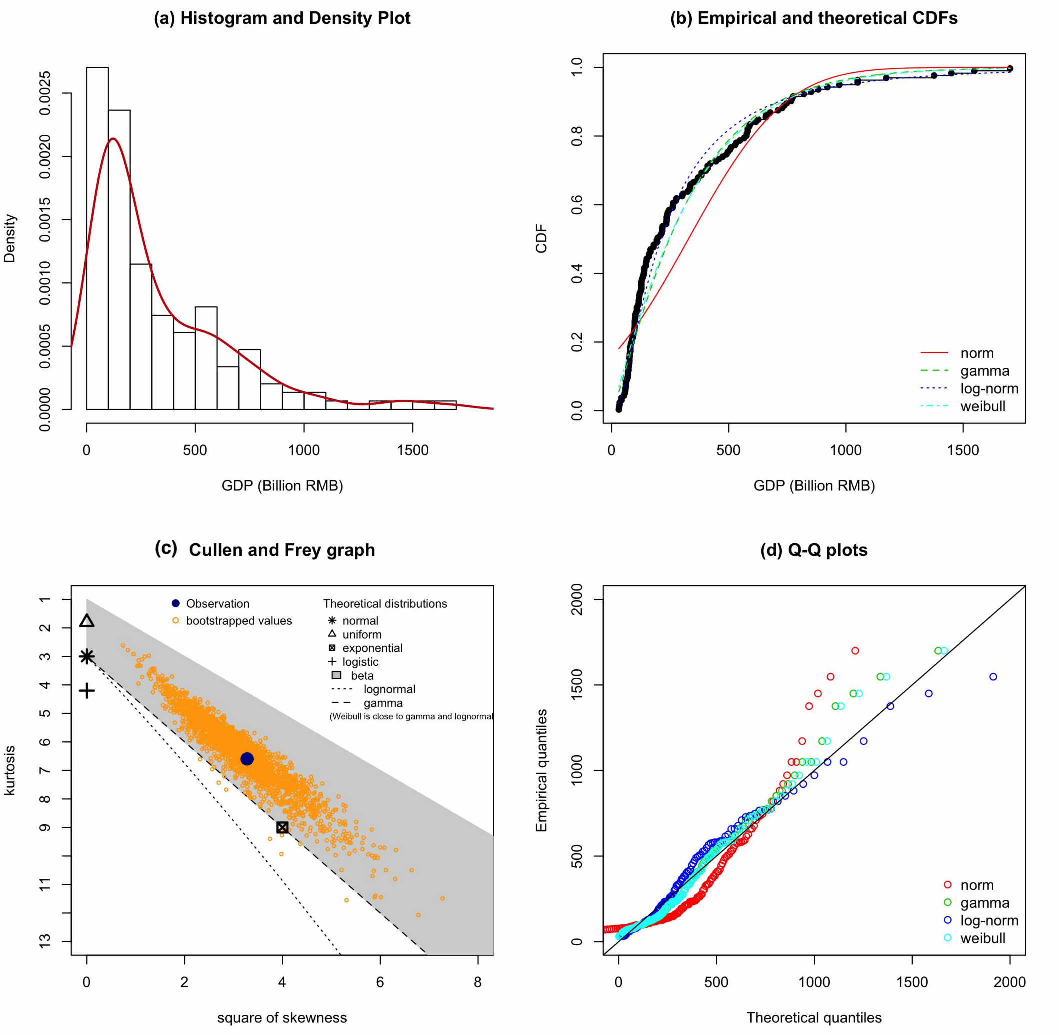

Figure S1. The empirical and theoretical distributions of all city GDP values. (a) the histogram of city GDPs; (b) the observed empirical cumulative distribution function (CDF) in black and four theoretical CDFs; (c) the Cullen and Frey graph shows the probability distribution fittings; (d) the Q-Q plot compares the empirical and theoretical quantile distributions.

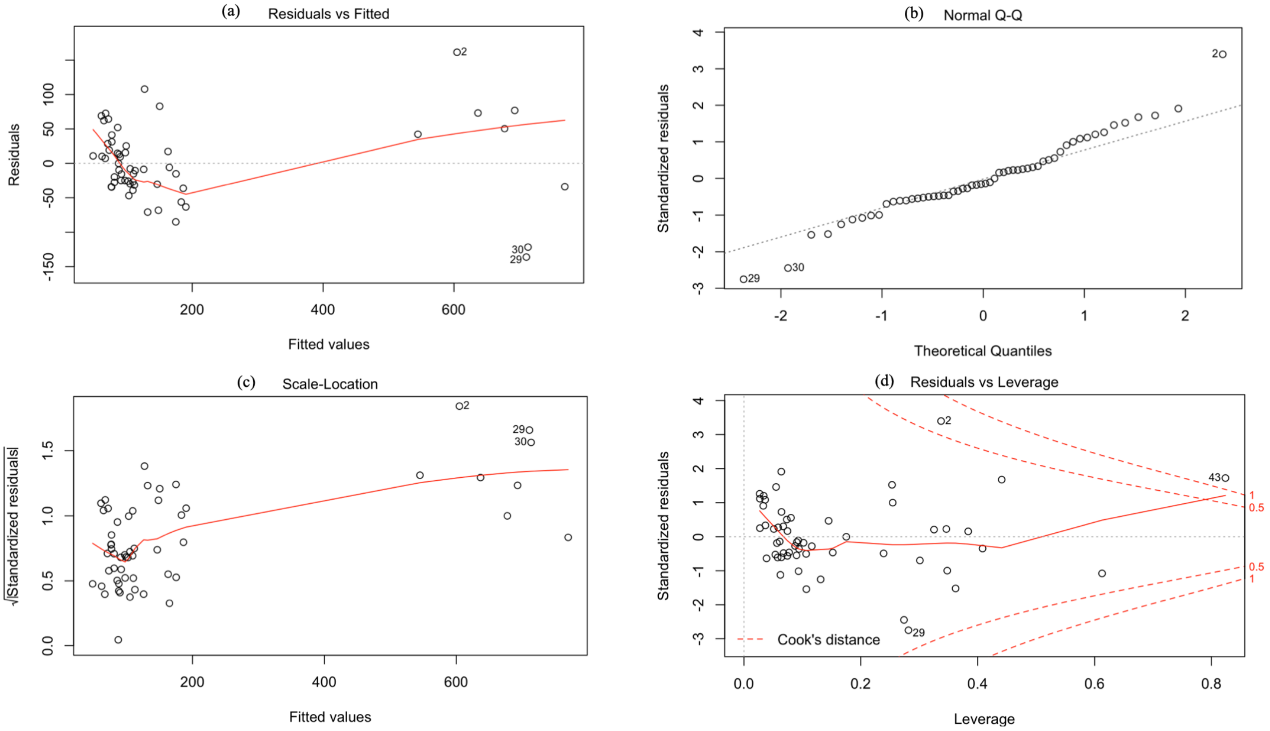

Figure S2. The residual plots of predicting city GDP values in Liaoning province using the multiple linear regression. (a) the scatter plot of residuals and fitted values; (b) the Normal Quantile-Quantile plot for standardized residuals; (c) the Scale-Location plot to check the residual spread; (d) the Residuals vs Leverage plot to find influential samples if any.

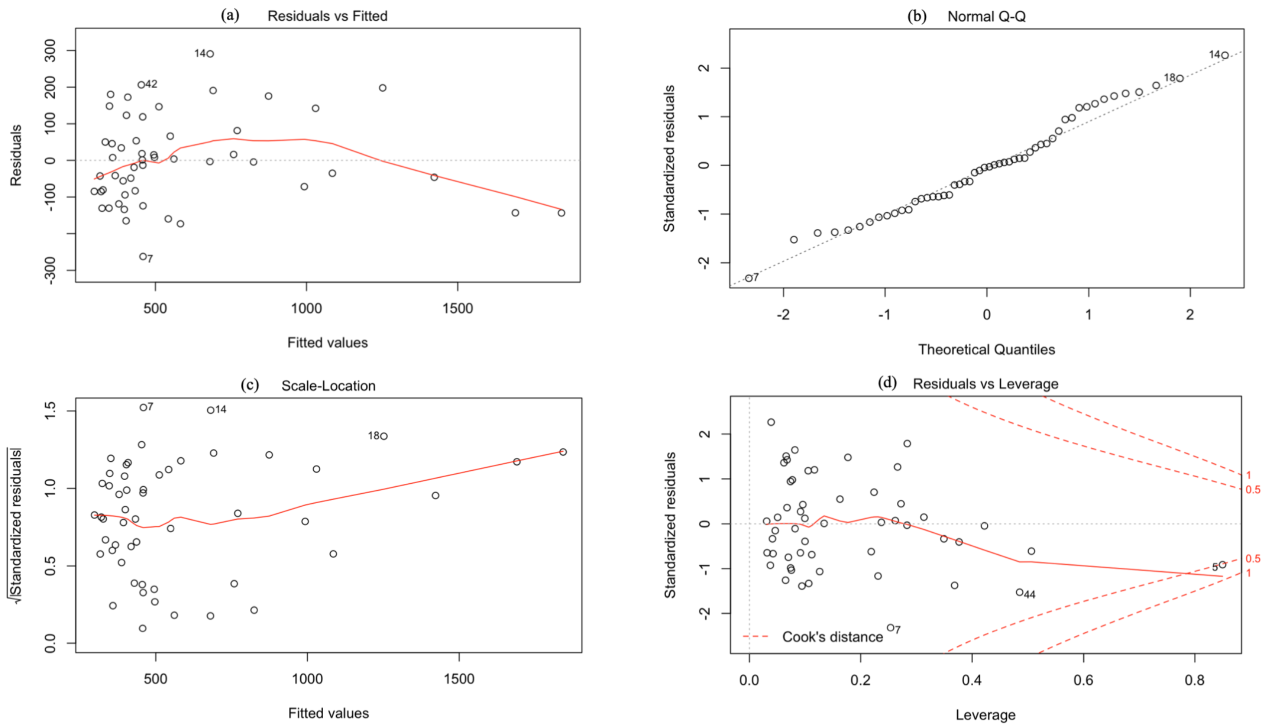

Figure S3. The residual plots of predicting city GDP values in Jiangsu province using the multiple linear regression. (a) the scatter plot of residuals and fitted values; (b) the Normal Quantile-Quantile plot for standardized residuals; (c) the Scale-Location plot to check the residual spread; (d) the Residuals vs Leverage plot to find influential samples if any.

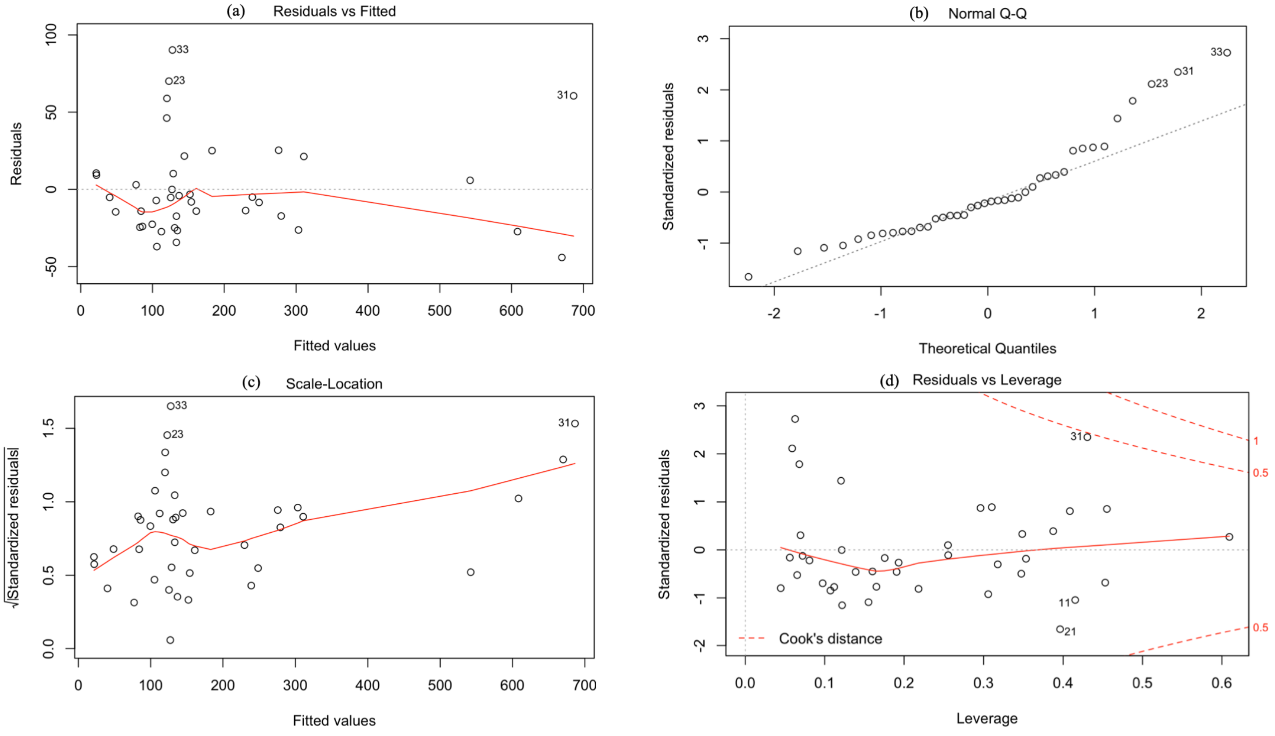

Figure S4. The residual plots of predicting city GDP values in Shaanxi province using the multiple linear regression. (a) the scatter plot of residuals and fitted values; (b) the Normal Quantile-Quantile plot for standardized residuals; (c) the Scale-Location plot to check the residual spread; (d) the Residuals vs Leverage plot to find influential samples if any.

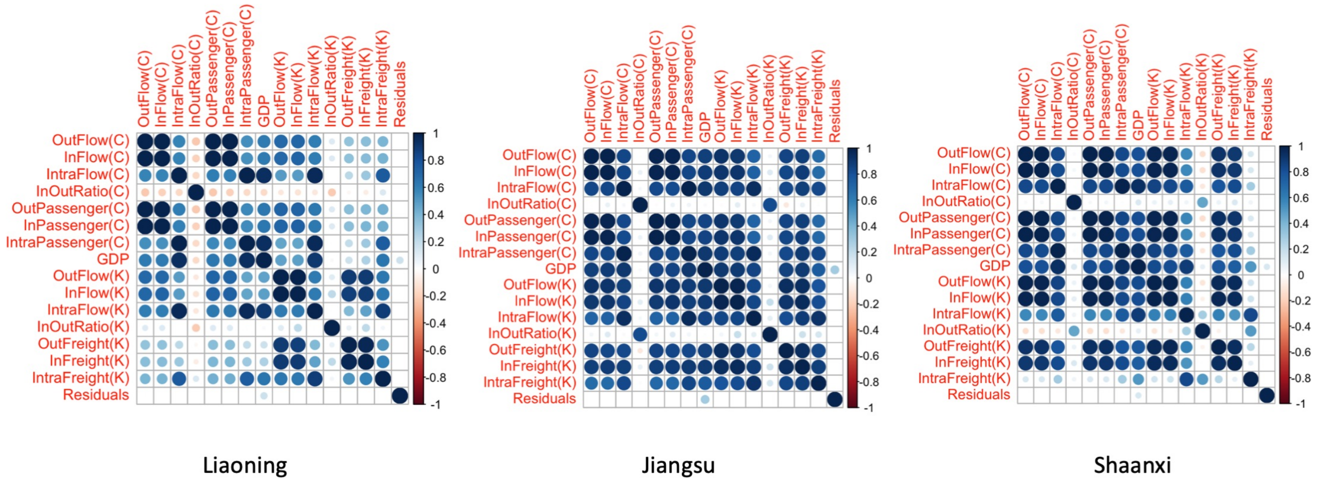

Figure S5. The visualization of correlation matrix of predictors and residuals in each province.

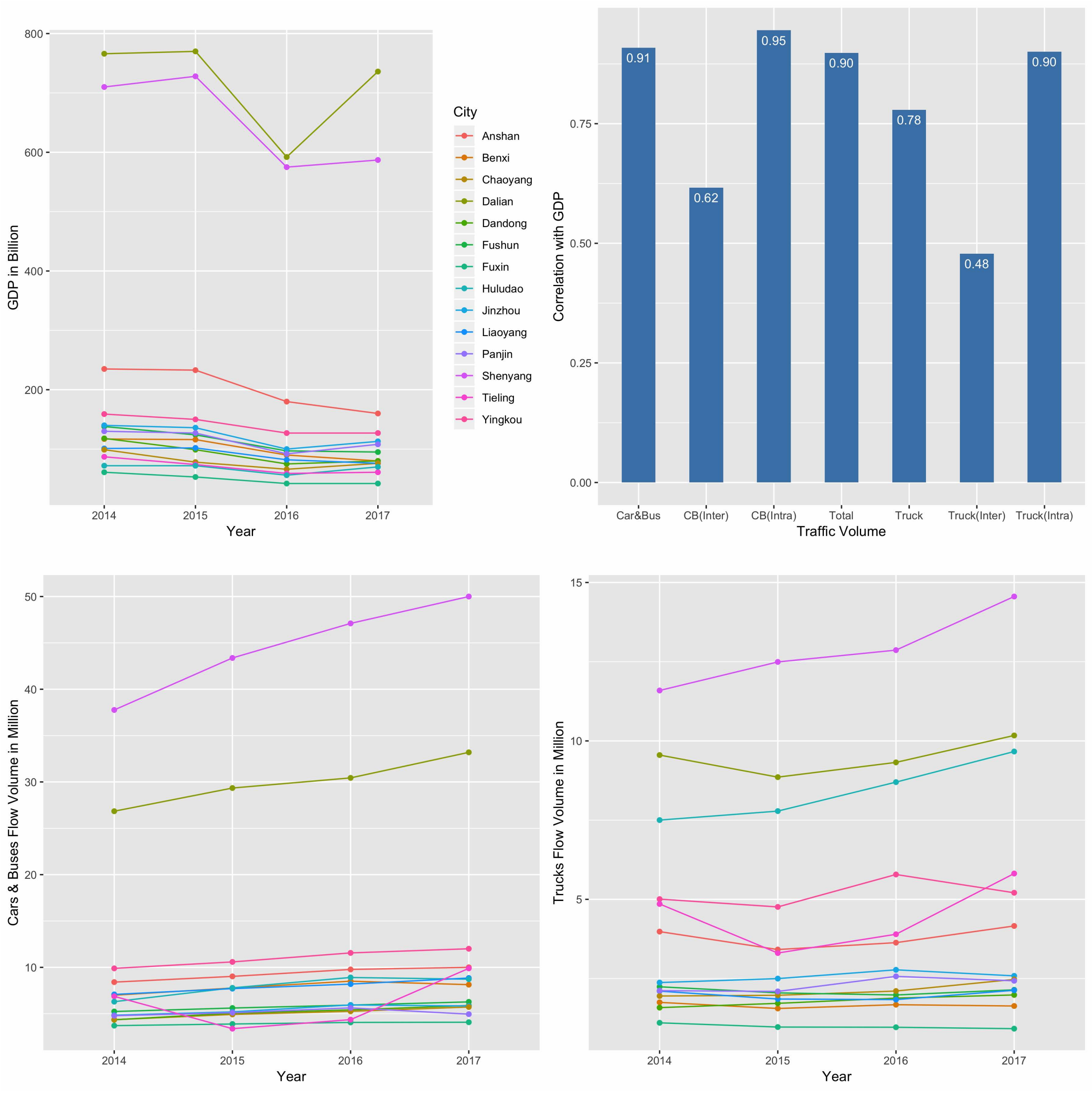

Figure S6. (a) The temporal changes of cities' GDP values; (b) the correlation between city GDP and traffic volumes; (c) the temporal changes of traffic volumes of cars and buses; (d) the temporal changes of traffic volumes of trucks in Liaoning province.

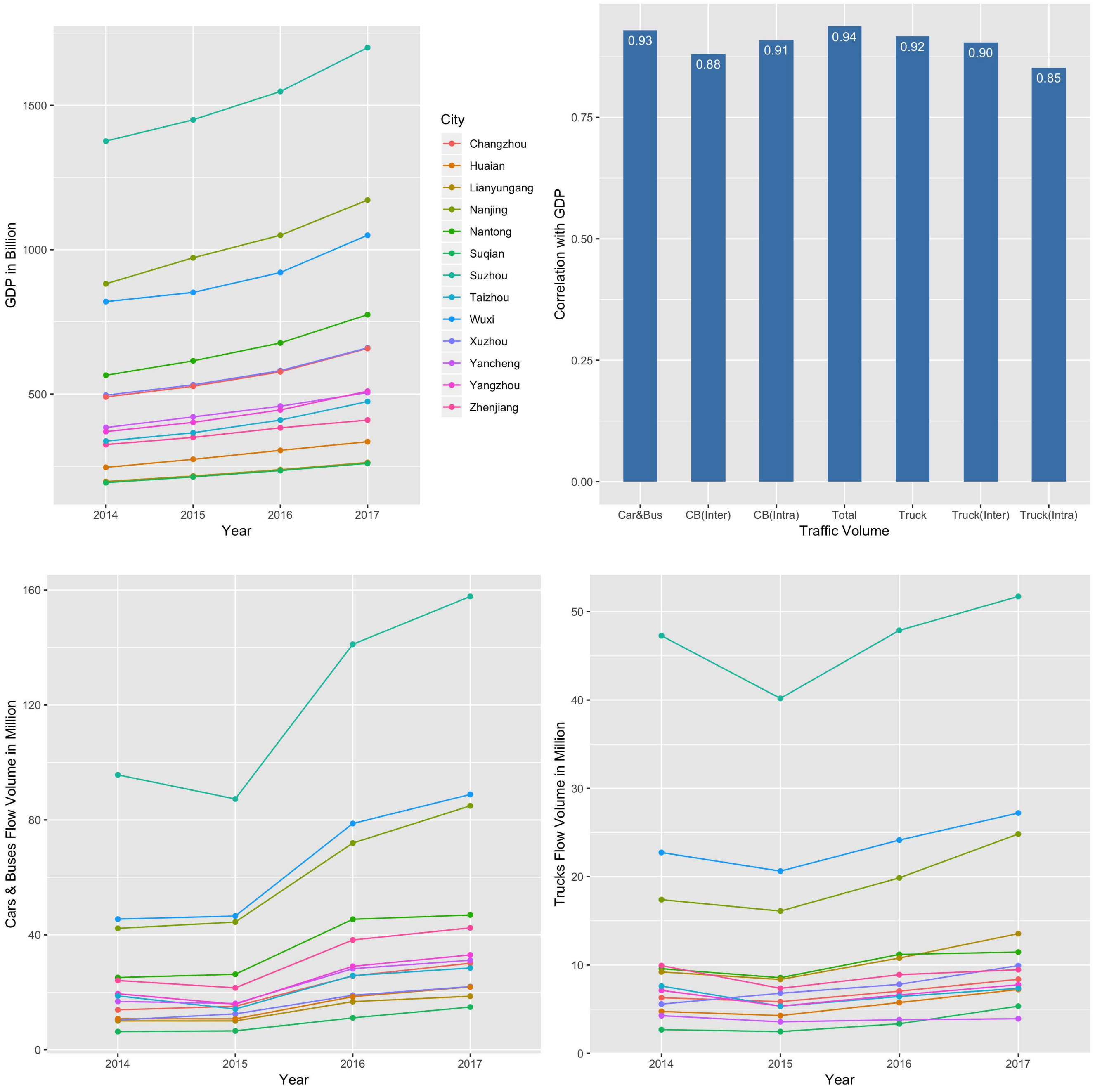

Figure S7. (a) The temporal changes of cities' GDP values; (b) the correlation between city GDP and traffic volumes; (c) the temporal changes of traffic volumes of cars and buses; (d) the temporal changes of traffic volumes of trucks in Jiangsu province.

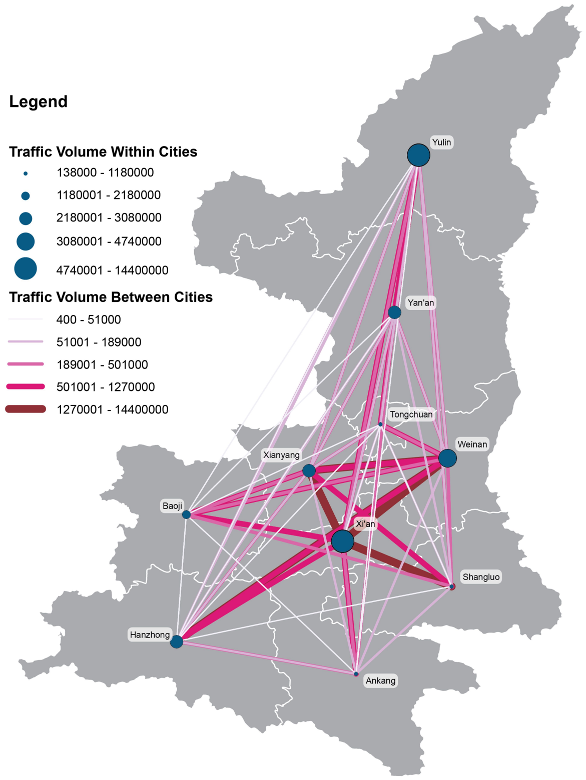

(a) 2014

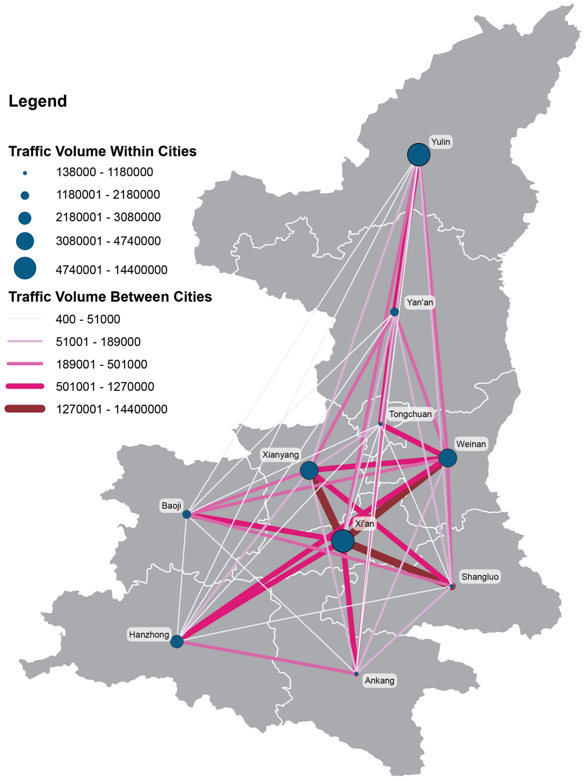

(b) 2015

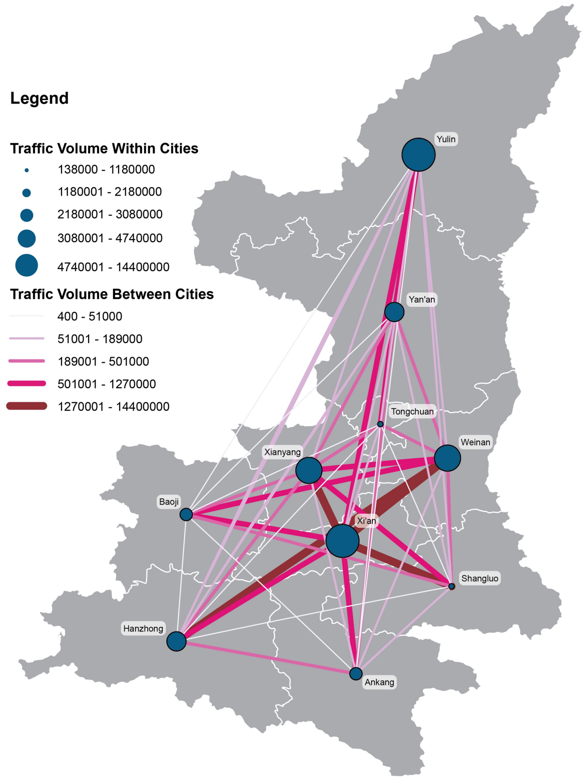

(c) 2016

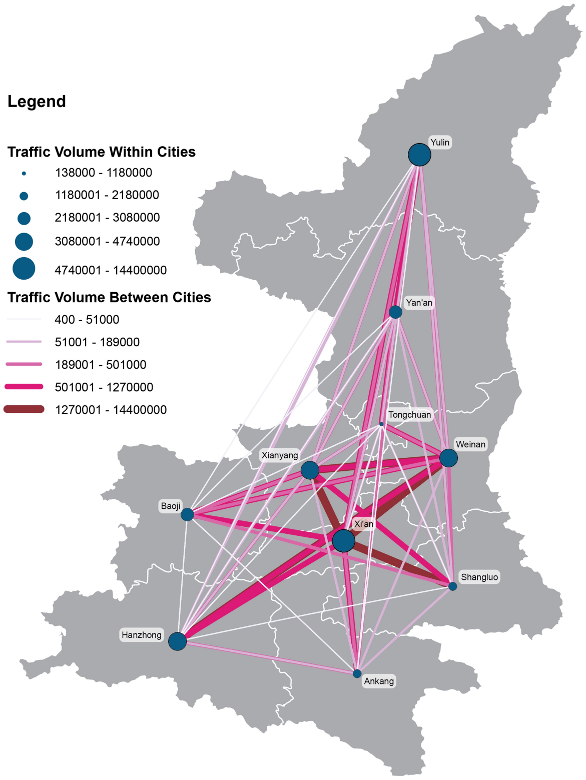

(d) 2017

Figure S8. Traffic Volume of Freight Trucks in Shaanxi. Note: The maps were generated using ArcMap version 10.6 and Adobe Illustrator CC version 20.

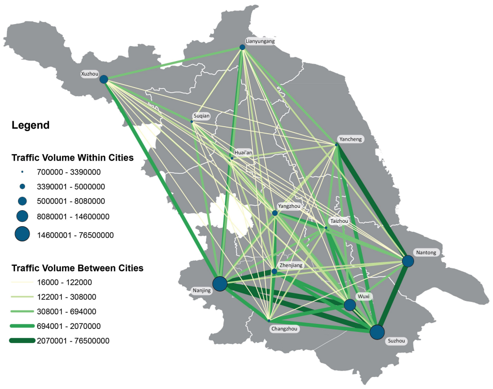

(a) 2014

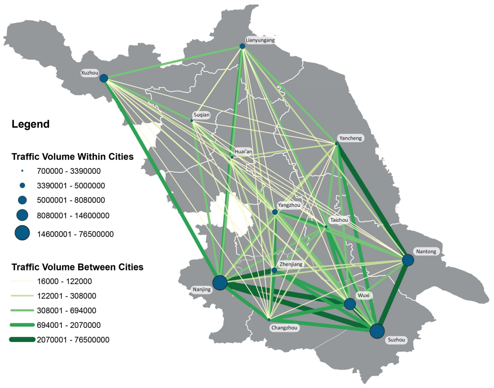

(b) 2015

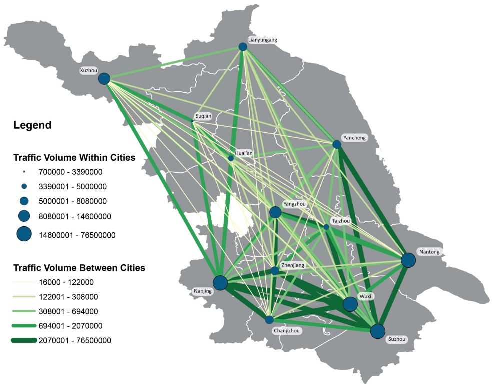

(c) 2016

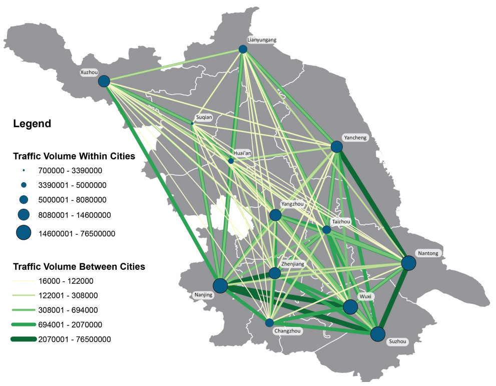

(d) 2017

Figure S9. Traffic Volume of Cars and Buses in Jiangsu. Note: The maps were generated using ArcMap version 10.6 and Adobe Illustrator CC version 20.

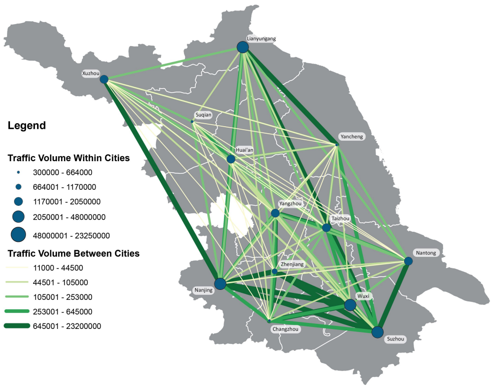

(a) 2014

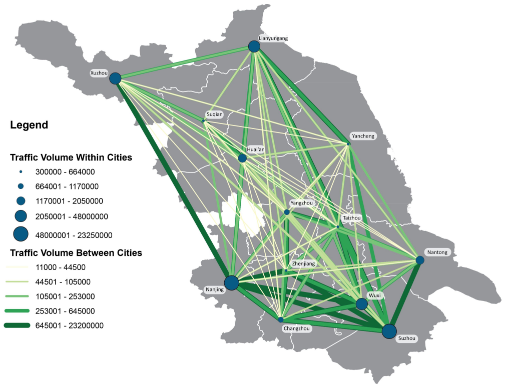

(b) 2015

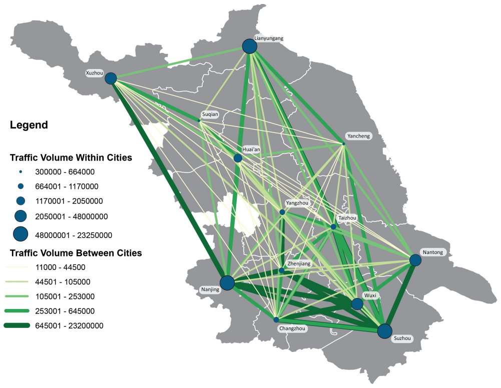

(c) 2016

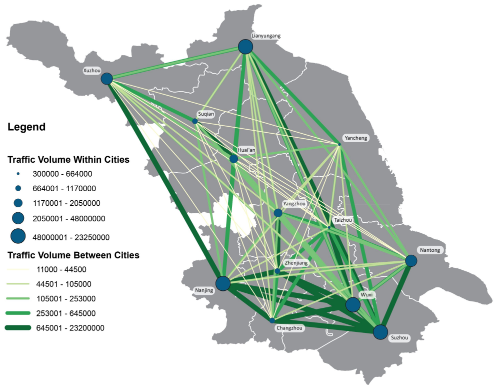

(d) 2017

Figure S10. Traffic Volume of Freight Trucks in Jiangsu. Note: The maps were generated using ArcMap version 10.6 and Adobe Illustrator CC version 20.

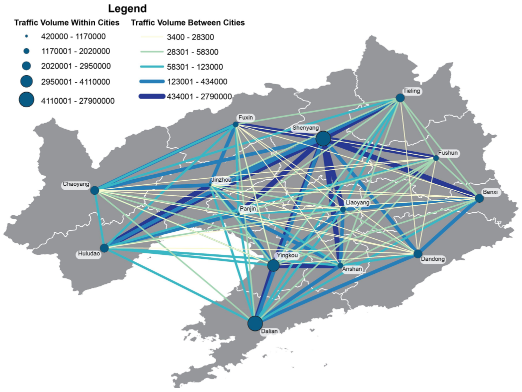

(a) 2014

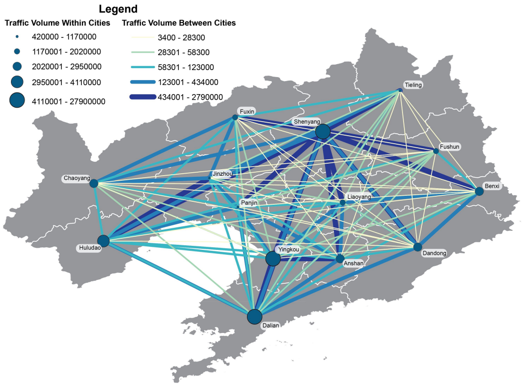

(b) 2015

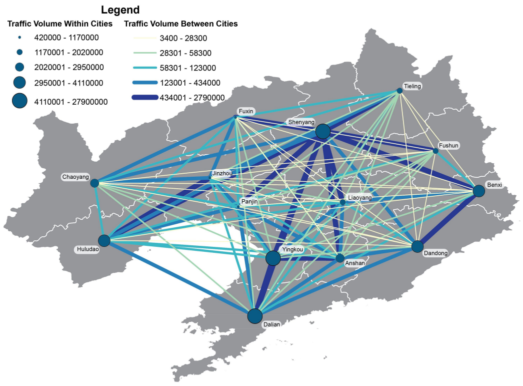

(c) 2016

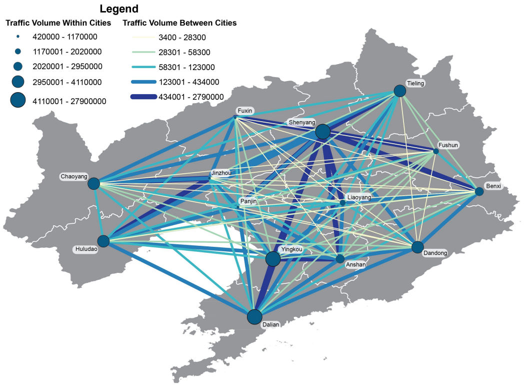

(d) 2017

Figure S11. Traffic Volume of Cars and Buses in Liaoning. Note: The maps were generated using ArcMap version 10.6 and Adobe Illustrator CC version 20.

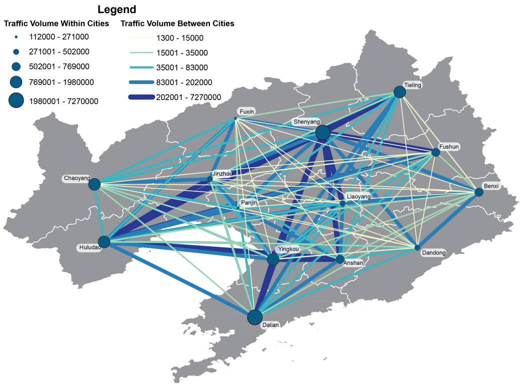

(a) 2014

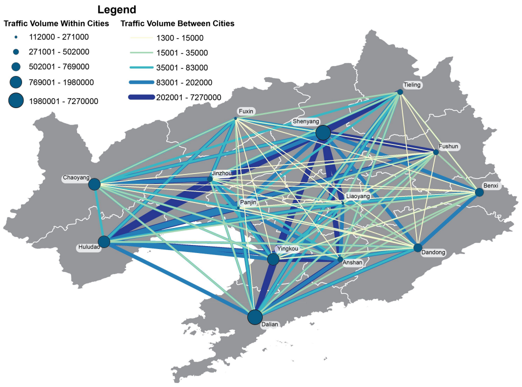

(b) 2015

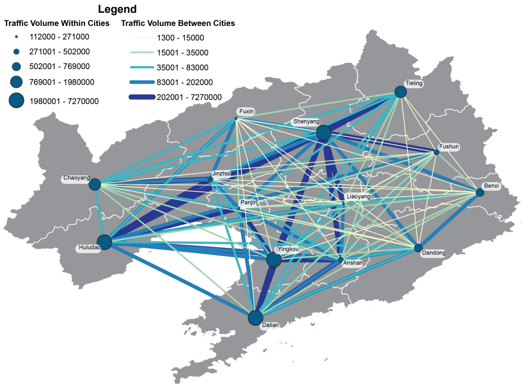

(c) 2016

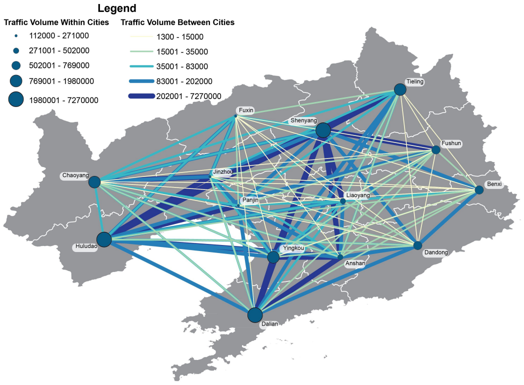

(d) 2017

Figure S12. Traffic Volume of Freight Trucks in Liaoning. Note: The maps were generated using ArcMap version 10.6 and Adobe Illustrator CC version 20.

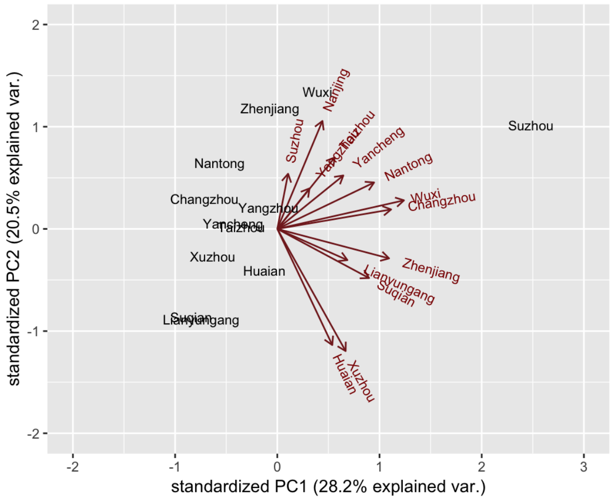

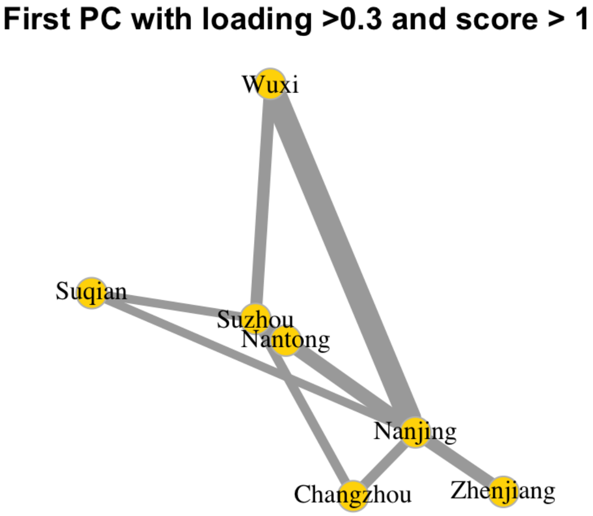

Figure S13. The PCA analysis results of spatial interaction networks of buses and cars in Jiangsu. Note: The figures were generated using RStudio version 1.2.

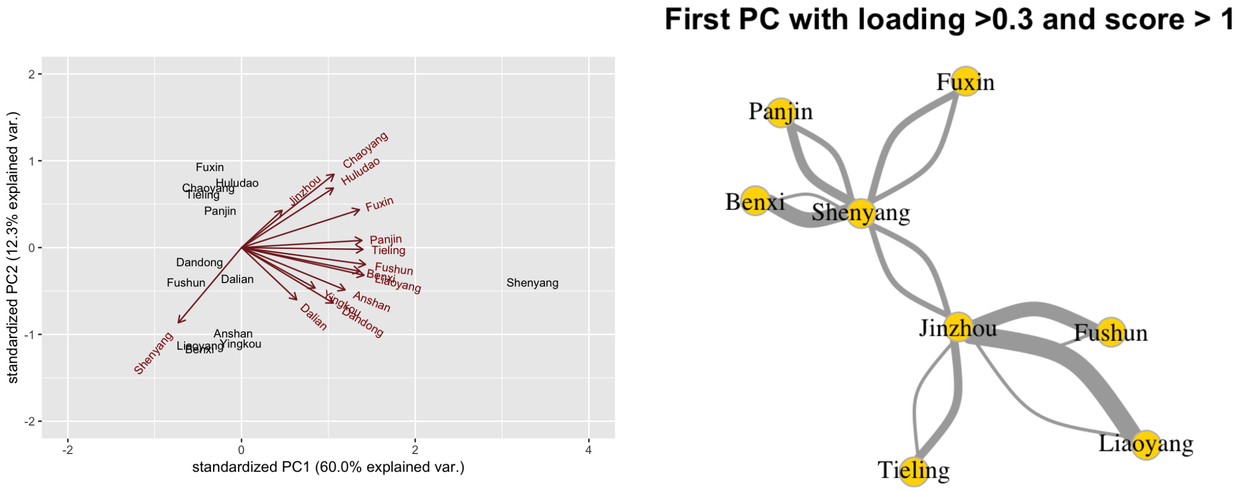

Figure S14. The PCA analysis results of spatial interaction networks of buses and cars in Liaoning. Note: The figures were generated using RStudio version 1.2.

Supplementary References:

71. Delignette-Muller, M. L. et al. fitdistrplus: An r package for fitting distributions. J. Stat. Softw. 64, 1–34 (2015).

72. Akaike, H. Information theory and an extension of the maximum likelihood principle. In Selected Papers of Hirotugu Akaike, vol. 1, 199–213 (Springer, 1998).

73. Cullen, A. C., Frey, H. C. & Frey, C. H. Probabilistic techniques in exposure assessment: A handbook for dealing with variability and uncertainty in models and inputs (Springer Science & Business Media, 1999).
